# Supplementary material for: Arpin Regulates Migration Persistence by Interacting with Both Tankyrases and the Arp2/3 Complex
Source: Int J Mol Sci. 2021 Apr 16;22(8):4115. doi: 10.3390/ijms22084115 (PMC8073056; doi:10.3390/ijms22084115)
Supplement: Supplementary file 1 [file ijms-22-04115-s001.zip › ijms-1166964-supplementary.pdf]

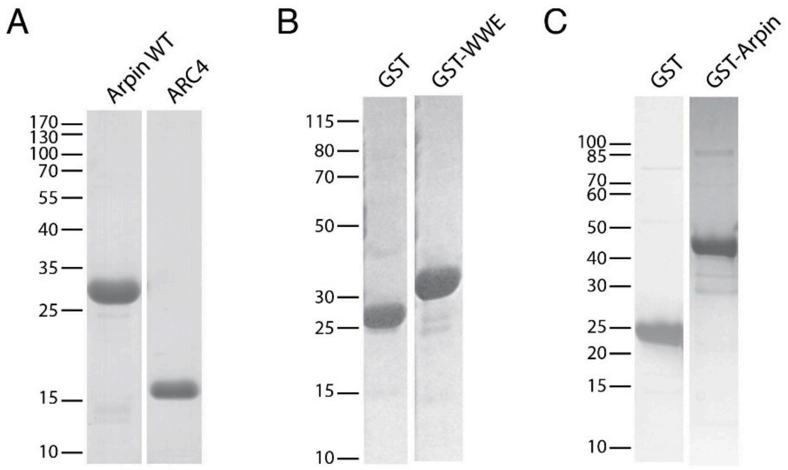

Figure S1. Purity of recombinant proteins used in the study. (**A**) Purified Arpin and ARC4 domain were used for Size Exclusion Chromatography – Multi Angle Light Scattering experiment of figure 1C. (**B**) Purified GST and GST-WWE were used for the pull-down assay of figure 2B. (**C**) Purified GST and GST-Arpin were used for the pull-down assay of figure 2E. Coomassis stained gels.

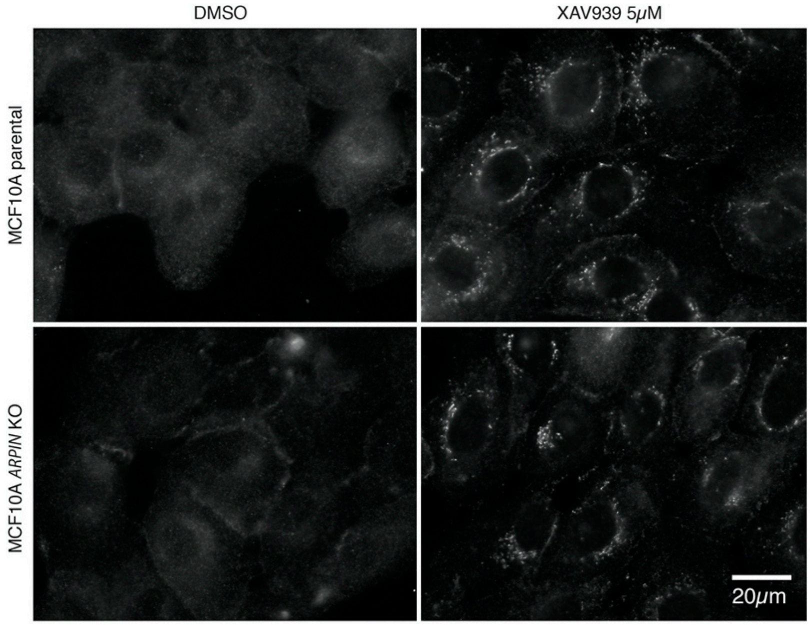

Figure S2. Immunofluorescence staining of TNKS in MCF10A WT and *ARPIN* KO cells treated with XAV939 at 5 μM for 9 h or with DMSO. TNKS are mostly cytosolic in both cases, but appear to condensate upon XAV939 treatment.

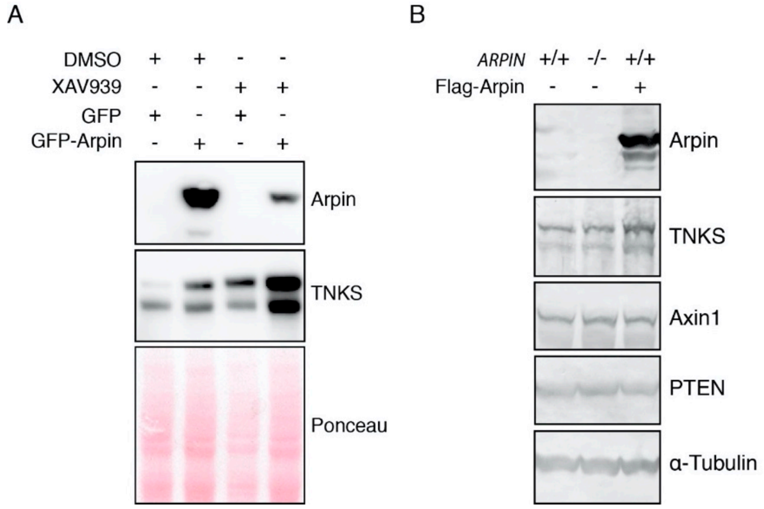

Figure S3. Arpin overexpression increases TNKS protein levels. (**A**) 293T cells were transiently transfected with GFP or GFP-Arpin and lysed. Where indicated, XAV939 1µM was added for 24 h (**B**) 293T WT or *ARPIN* KO cells were transiently transfected with indicated constructs and lysed. Protein levels were revealed by Western blot with corresponding antibodies.

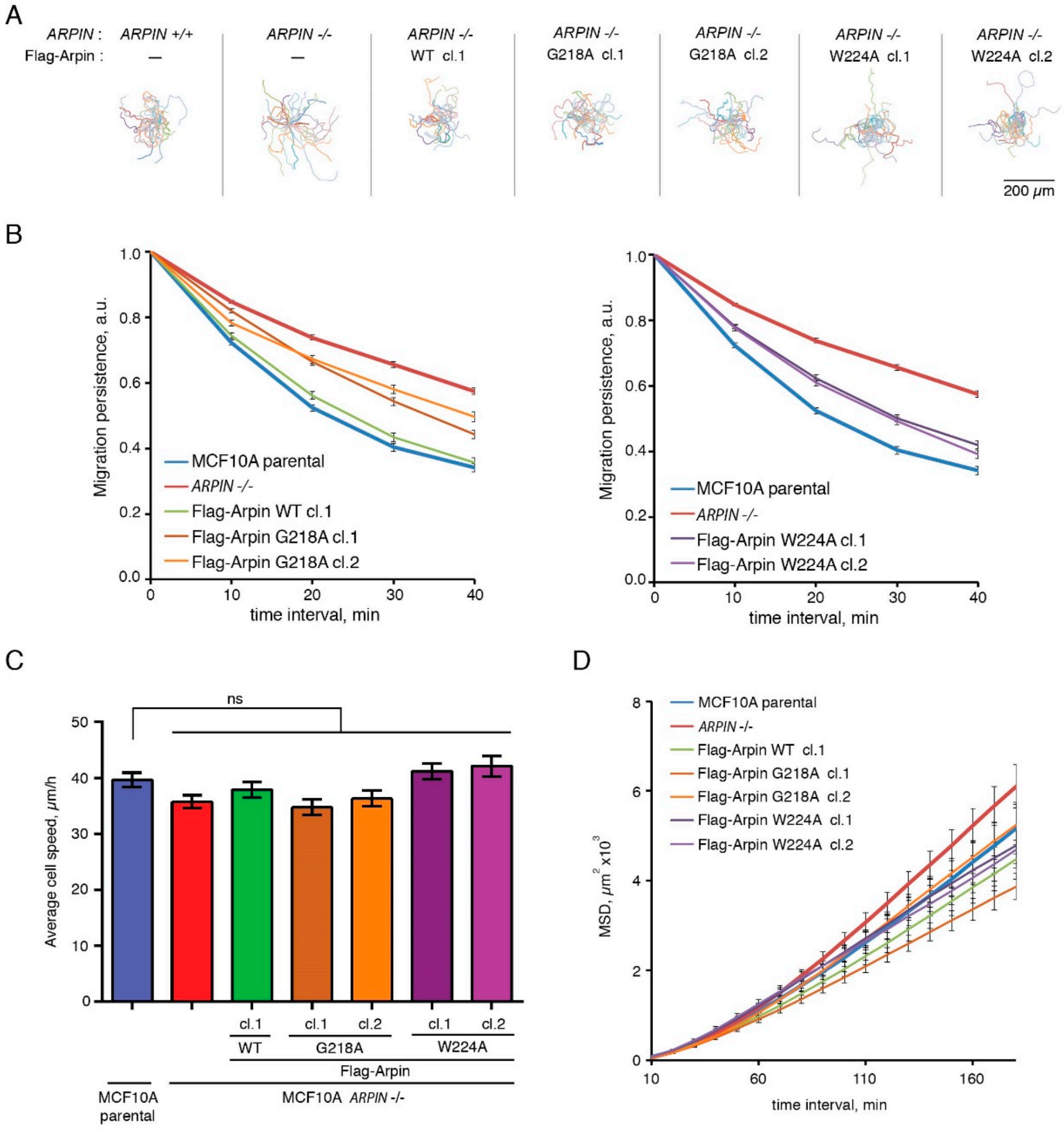

Figure S4. Single cell migration assay of stable MCF10A clones expressing WT, G218A and W224A Flag-Arpin. (**A**) Single cell trajectories (25 cells tracked for 7 h displayed. (**B**) Migration persistence displayed here for the whole cell population as an autocorrelation curve [1]. (**C**) Average cell speed (**D**) Mean square displacement (MSD) is represented as its mean value and standard error of the mean (number of cells ranges from 41 to 80).

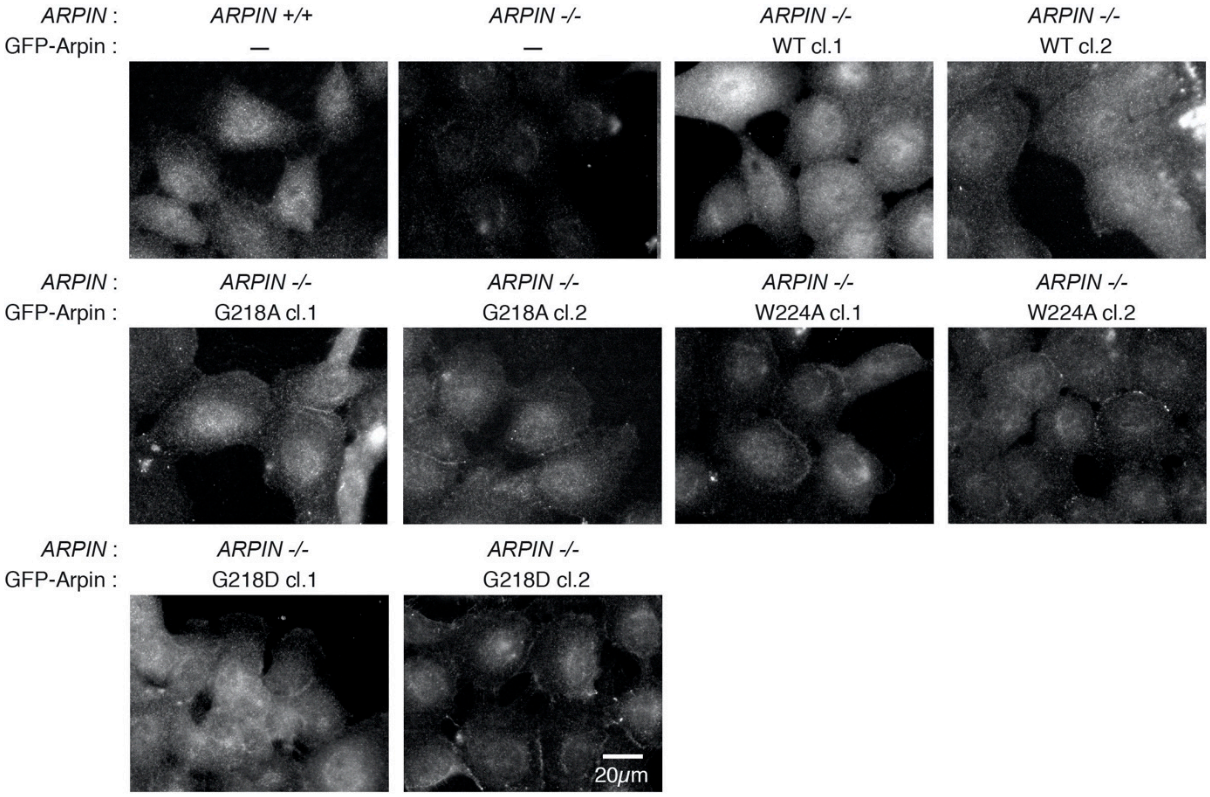

Figure S5. Immunofluorescence of Arpin in MCF10A parental cells, ARPIN knock-out and knock-in clones. All Arpin forms were localized in the cytosol and nucleus with an occasional peripheral staining, which does not systematically correspond either to cell junctions or to free edges.

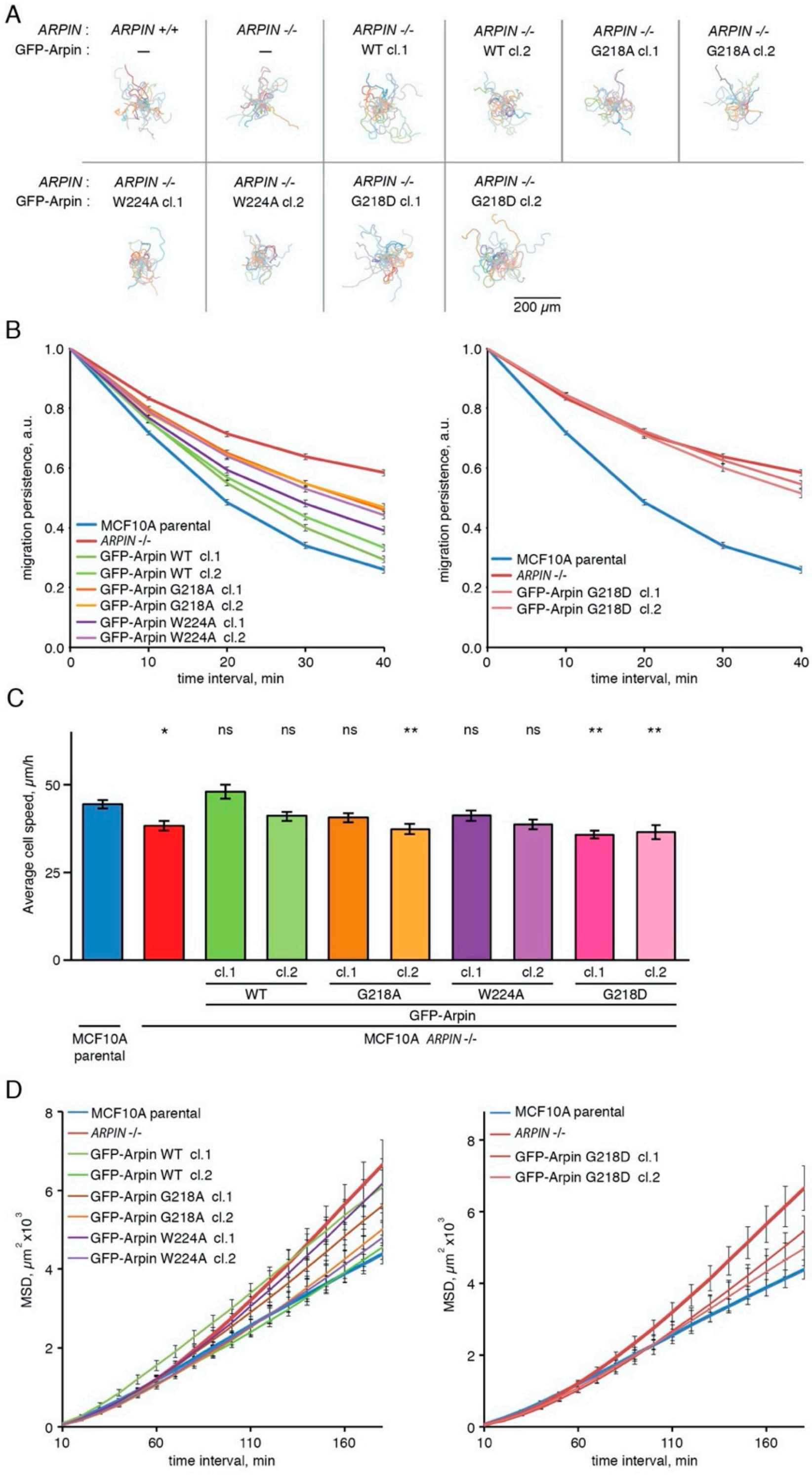

Figure S6. Single cell migration assay of *ARPIN* knock-in MCF10A clones. (**A**) Single cell trajectories (25 cells tracked for 7 h displayed). (**B**) Migration persistence displayed here for the whole cell population as an autocorrelation curve [1]. (**C**) Average cell speed (Kruskal-Wallis comparison with control MCF10A cells, * $p < 0.05$, ** $p < 0.01$, *** $p < 0.001$). (**D**) Mean square displacement (MSD) is represented as its mean value and standard error of the mean (number of cells ranges from 47 to 83).

References:

1. Gorelik, R.; Gautreau, A. Quantitative and Unbiased Analysis of Directional Persistence in Cell Migration. *Nature Protocols* **2014**, *9*, 1931–1943, doi:10.1038/nprot.2014.131.
